# Elevated *HLA-A* expression impairs HIV control through inhibition of NKG2A-expressing cells

**DOI:** 10.1126/science.aam8825

**Published:** 2018-01-04

**Authors:** Veron Ramsuran, Vivek Naranbhai, Amir Horowitz, Ying Qi, Maureen P. Martin, Yuko Yuki, Xiaojiang Gao, Victoria Walker-Sperling, Gregory Q. Del Prete, Douglas K. Schneider, Jeffrey D. Lifson, Jacques Fellay, Steven G. Deeks, Jeffrey N. Martin, James J. Goedert, Steven M. Wolinsky, Nelson L. Michael, Gregory D. Kirk, Susan Buchbinder, David Haas, Thumbi Ndung’u, Philip Goulder, Peter Parham, Bruce D. Walker, Jonathan M. Carlson, Mary Carrington

**Affiliations:** 1 Cancer and Inflammation Program, Leidos Biomedical Research, Inc., Frederick National Laboratory for Cancer Research, Frederick, MD 21702, USA; 2 Ragon Institute of Massachusetts General Hospital, Massachusetts Institute of Technology and Harvard University, Cambridge, MA 02139, USA; 3 KwaZulu-Natal Research Innovation and Sequencing Platform (KRISP), School of Laboratory Medicine and Medical Sciences, University of KwaZulu-Natal, Durban, South Africa; 4 Centre for the AIDS Programme of Research in South Africa (CAPRISA), Durban, South Africa; 5 Wellcome Trust Centre for Human Genetics, Nuffield Department of Medicine, University of Oxford, Oxford, UK; 6 Department of Oncological Sciences, Precision Immunology Institute, Icahn School of Medicine at Mount Sinai, New York, NY 10029, USA; 7 Cancer and Inflammation Program, Center for Cancer Research, National Cancer Institute, National Institutes of Health, Frederick, MD 21702, USA; 8 AIDS and Cancer Virus Program, Leidos Biomedical Research, Inc., Frederick National Laboratory for Cancer Research, Frederick, MD 21702, USA; 9 School of Life Sciences, École Polytechnique Fédérale de Lausanne, and Swiss Institute of Bioinformatics, Lausanne, Switzerland; 10 Department of Medicine University of California, San Francisco, CA 94143, USA; 11 Department of Epidemiology and Biostatistics, University of California, San Francisco, CA 94143, USA; 12 Infections and Immunoepidemiology Branch, Division of Cancer Epidemiology and Genetics, National Cancer Institute, National Institutes of Health, Rockville, MD 20850, USA; 13 Division of Infectious Diseases, The Feinberg School of Medicine, Northwestern University, Chicago, IL 60611, USA; 14 U.S. Military HIV Research Program, Walter Reed Army Institute of Research, Silver Spring, MD 20910, USA; 15 Department of Epidemiology, Johns Hopkins University Bloomberg School of Public Health, Baltimore, MD 21205, USA; 16 San Francisco Department of Public Health, HIV Research Section, San Francisco, CA 94102, USA; 17 Vanderbilt University School of Medicine, Nashville, TN 37232, USA; 18 African Health Research Institute, Durban, South Africa; 19 HIV Pathogenesis Programme, Doris Duke Medical Research Institute, Nelson R. Mandela School of Medicine, University of KwaZulu-Natal, Durban, South Africa; 20 Max Planck Institute for Infection Biology, Berlin, Germany; 21 Department of Paediatrics, University of Oxford, Oxford, UK; 22 Departments of Structural Biology and Microbiology and Immunology, Stanford University, Stanford, CA 94305, USA; 23 Institute for Medical and Engineering Sciences, Massachusetts Institute of Technology, Cambridge, MA 02139, USA; 24 Microsoft Research, Redmond, WA 98052, USA

## Abstract

The highly polymorphic human leukocyte antigen (*HLA*) locus encodes cell surface proteins that are critical for immunity. *HLA-A* expression levels vary in an allele-dependent manner, diversifying allele-specific effects beyond peptide-binding preference. Analysis of 9763 HIV-infected individuals from 21 cohorts shows that higher *HLA-A* levels confer poorer control of HIV. Elevated *HLA-A* expression provides enhanced levels of an HLA-A–derived signal peptide that specifically binds and determines expression levels of HLA-E, the ligand for the inhibitory NKG2A natural killer (NK) cell receptor. *HLA-B* haplotypes that favor NKG2A-mediated NK cell licensing (i.e., education) exacerbate the deleterious effect of high *HLA-A* on HIV control, consistent with NKG2A-mediated inhibition impairing NK cell clearance of HIV-infected targets. Therapeutic blockade of HLA-E:NKG2A interaction may yield benefit in HIV disease.

Diversity within regions of human leukocyte antigen (HLA) class I molecules that determine peptide-binding specificity has a major impact on human disease pathogenesis. Variation in expression levels across alleles of certain *HLA* genes has also been shown to associate with disease outcome (1–6), emphasizing the importance of *HLA* polymorphism that determines characteristics other than peptide specificity alone. Elevated expression levels of HLA-C associates with reduced HIV viral load (VL) (1), resulting, in part, from a greater frequency of cytotoxic T lymphocyte (CTL) responses to HLA-C–restricted peptides with increasing HLA-C. Like *HLA-C, HLA-A* alleles vary in expression levels in an allotype-specific manner (7), but these two class I loci have many distinguishing characteristics. Compared with HLA-C, HLA-A is expressed at a 13- to 18-fold higher level on the cell surface (8) and is about twofold more polymorphic. Mechanisms of transcriptional regulation for these two loci are also distinct under healthy conditions (7, 9, 10). These and other differences may affect how these two loci affect human disease.

We verified that the pattern of allele-specific variation in *HLA-A* expression levels was not modified by HIV infection by comparing *HLA-A* expression in 243 HIV-uninfected and 162 HIV-infected ethnicity-matched individuals (fig. S1). Being HIV infected did not associate with a change in the overall level of *HLA-A* mRNA expression (Effect_unadjusted_ = 0.00, SE = 0.07, *P* =1), nor did HIV status modify expression estimates for any single *HLA-A* allele (interaction *P*-values were 0.226 to 0.987 for each of the alleles tested). Therefore, in HIV infection, the gradient in *HLA-A* expression level attributable to each allele is similar to that in healthy individuals.

To test whether *HLA-A* expression levels are associated with HIV control, we examined a pooled data set of 2298 HIV-infected (clade C) individuals recruited at 11 sites in sub-Saharan Africa, in which the estimated effect of each *HLA* allele on HIV VL measured cross-sectionally has been reported (11). The *HLA-A* expression level of each allele, estimated for black African individuals, was positively correlated with the estimate of effect of that allele on HIV VL (correlation coefficient *R* = 0.54, *P* = 0.007, Fig. 1A and Table 1).

Next, we sought to validate the discovery of a deleterious effect of elevated *HLA-A* expression level in independent cohorts with prospective follow-up and of broader demographic background. We included 62,843 VL measurements obtained longitudinally over a total of 32,804 person years of antiretroviral therapy–free observation time (median 2.86 years per individual) in 5818 individuals enrolled in one of six studies in the USA or one study in Switzerland (see online methods). We modeled *HLA-A* expression as *z*-scores (equivalent to one standard deviation change in expression level), using mRNA levels measured in 436 white and black healthy donors (table S1). Consistent with the discovery analysis among sub-Saharan Africans, elevated *HLA-A* expression levels were significantly associated with higher HIV viremia, even after accounting for the individual allelic effects of *HLA-A, -B*, and -*C*. For every one *z*-score increase in *HLA-A* expression level, the VL increase over time was 0.06 log_10_ copies/ml higher (*P* = 4.4 × 10^−19^; Table 1). Grouping individuals by estimated *HLA-A* expression level demonstrates the effect of increasing *HLA-A* expression on unadjusted HIV VL (Fig. 1B).

The association between *HLA-A* expression level and HIV viremia was independently significant in each ethnicity stratum (*P*_whites_ = 6.1 × 10^−6^; *P*_Africans/African-Americans_ = 1.1 × 10^−18^; and *P*_Hispanic/other_ = 2.3 × 10^−10^), notwithstanding distinct *HLA-A* allelic frequencies in each ethnic group. Among 2019 donors enrolled during acute, early HIV infection with known dates of seroconversion, elevated *HLA-A* expression was similarly associated with higher VL (*P* = 2.5 × 10^−9^), confirming that this finding is unlikely to be confounded by frailty bias. *HLA-A* expression level was associated with a spectrum of alternative HIV outcomes, including elevated mean VL (*P* = 9.3 × 10^−12^) and odds of being an HIV noncontroller (HIV VL >10,000 copies/ml) relative to being a controller (HIV VL <2000 copies/ml) (*P* = 9.2 × 10^−11^). Furthermore, among 2100 individuals for whom longitudinal CD4^+^ T cell count measures were available, higher *HLA-A* expression was strongly, and substantially, associated with reduced CD4^+^ T cell counts (Table 1). The effects of *HLA-A* expression levels on VL and CD4 count were stable over time (Fig. 1, B and C), consistent with a temporally sustained mechanism. Finally, we examined a partially nonoverlapping (39.1% of donors were not included in the VL analyses) collection of five natural-history cohorts, including 1159 antiretroviral-naïve individuals followed prospectively after HIV infection. Even in this limited sample, elevated *HLA-A* expression was associated with accelerated progression to AIDS_1987_ (*P* = 0.04) and progression to CD4^+^ T cell count of <200 cells/µl (*P* = 0.02), again after adjusting for all individual HLA alleles.

*HLA-A* expression levels vary across alleles in a continuous manner, indicating multiple polymorphic regulatory sites that together determine the expression level of any given allele. As no single variant controls *HLA-A* expression levels, genome-wide association studies (GWAS) are not expected to detect such effects. Using formal *HLA-A* typing results, we inferred expression level for 3057 white, Hispanic, and black individuals included in the International HIV Controllers GWAS (12) (40% of whom were not included in any of the analyses described above). *HLA-A* expression was significantly associated with HIV elite controller or noncontroller status even after adjusting for population structure (*P* = 2.7 × 10^−5^). This observation emphasizes a limitation of GWAS when the combined effects of multiple genetic variants determine a phenotype.

Next, we sought to determine the likely mechanism(s) for the finding that elevated *HLA-A* expression associates with impaired HIV control. HLA-E serves as a ligand for the strongly inhibitory receptor CD94/NKG2A expressed on both natural killer (NK) cells and T cells. Expression of HLA-E is dependent on stable binding of a signal peptide derived from the leader sequence of HLA-A, -B and -C molecules (residues −22 to −14 relative to the mature protein) (13, 14). Methionine at position 2 of the signal peptide (residue −21) stabilizes and promotes HLA-E expression, and all HLA-A and -C allotypes are fixed for methionine, whereas *HLA-B* contains a polymorphism that encodes either methionine (−21M) or threonine (−21T) at this position (15, 16). Unlike *HLA-A*, there is minimal variance in *HLA-B* transcriptional levels across alleles and individuals (17), so HLA-E expression is expected to vary not as a consequence of differences in *HLA-B* expression levels, but rather as a result of HLA-B −21M/T variation. Accordingly, HLA-B −21M enhances HLA-E expression level in a copy-dependent manner (15). We tested whether *HLA-A* expression levels may similarly be associated with HLA-E expression levels. Among 58 healthy donors, higher predicted *HLA-A* expression levels, and therefore higher HLA-A–derived signal peptide, was significantly correlated with higher HLA-E expression levels on the cell surface, independently of the reported effects of HLA-B −21 (Fig. 2A and table S2).

*HLA-E* has two common allelic variants denoted E*01:01 and E*01:03, reportedly varying in peptide affinities, peptide repertoires, and surface expression levels (18). Although HLA-E*01:03 associates with higher surface expression in univariate analyses, this association was not significant after adjusting for *HLA-B* −*21* and *HLA-A* genotypes (table S2). As HLA-E*01:03 and *HLA-B* −*21M* alleles are in significant linkage disequilibrium (*D*′ = 0.52), the increased peptide supply attributable to HLA-B −21M and HLA-A expression level likely account for higher expression of HLA-E* 01:03, rather than the variant distinguishing HLA-E*01:03 from – E*01:01. Accordingly, *HLA-E* variants did not show independent association with HIV outcomes (table S3). Similarly, addition of *HLA-E* genotype to a model fitting *HLA-A* expression and *HLA-B* −*21M* (and their interaction) was inferior to a model excluding *HLA-E* genotype in explaining HIV viremia.

The responsiveness of NK cells varies according to the presence of inhibitory-receptor/HLA pairs because of a process termed NK cell education or licensing (19). Accordingly, quantitative variation in HLA expression may influence target cell recognition through both ligand density variation and licensing modulation. The *HLA-B* −*21 M/T* variant distinguishes between two sets of *HLA* haplotypes that have differential effects on NK cell education, where −*21M* marks haplotypes that bias toward NKG2A-mediated education and −*21T* marks alternative haplotypes that bias toward KIR (killer cell immunoglobulin-like receptor)–mediated education (15). The reported linkage disequilibrium between *HLA-B* −*21M* and *HLA-B Bw6/HLA-C* group1 alleles that interact poorly with KIR is evident in our cohort (fig. S2). Using a ligand-independent activation assay designed to measure NK cell licensing, NKG2A^+^/KIR^−^ NK cells from *HLA-B* −*21MM*+ donors were more responsive than NKG2A^−^/KIR^+^ NK cells from the same donors (*P*_wilcoxon_ = 1.5 × 10^−6^), and notably, the strength of licensing among NKG2A^+^/KIR^−^ NK cells correlated with *HLA-A* expression level (*R* = 0.69, *P* = 0.03; Fig. 2B). Conversely, KIR^+^/ NKG2A^−^ NK cells were more strongly licensed in HLA-B −21TT donors (*P* = 1.1 × 10^−5^), and this was not correlated with *HLA-A* expression. Thus, *HLA* haplotypes characterized by both *HLA-B* −*21M* and high *HLA-A* genotypes, which provide highest levels of HLA-E epitope, strongly bias toward NKG2A-mediated education.

We next tested whether variation in *HLA-A* expression alters NK cell responses toward HIV-infected target cells, and whether this varies according to *HLA-B* −*21* genotype. Increasing *HLA-A* expression was significantly correlated with greater inhibition of NK cell degranulation exclusively among HLA-B −21MM donors, when target cells were HIV infected and the autologous effector NK cells necessarily expressed NKG2A (*R* = −0.77, *P* = 0.016, Fig. 2C). These data extend previous observations (20).

We reasoned that the genetic epidemiological effect of *HLA-A* expression level on impairing HIV control may vary according to *HLA-B* −*21* genotype. We examined the two extremes in variation of NK cell education demarcated by *HLA-B* −*21* MM versus TT, although education varies across a continuum(21). Haplotypes tagged by *HLA-B* −*21M* exacerbate the deleterious effect of *HLA-A* expression on HIV viremia (interaction *P*= 5.3×10^−9^), regardless of ethnicity (Fig. 3). The effect of *HLA-A* expression level on HIV viremia is of greater magnitude in individuals with two *HLA-B* methionine-encoding alleles [VL^effect-MM^ = 0.22, 95% confidence interval (CI) 0.17–0.26 log_10_ copies/ml per one *z*-score, *P* = 1.5 × 10^−21^] than in donors with two threonine-encoding *HLA-B* alleles (VL_effect-TT_ = 0.06, 95% CI 0.04–0.08 log_10_ copies/ml per one *z*-score, *P* = 1.8 × 10^−9^). The independent effect of *HLA-B* −*21M* varied across Caucasians and Africans/African Americans (fig. S4), perhaps owing to substantial differences in *HLA* haplotypes in Africans. In an *HLA-B* −21M/M individual, decrease in *HLA-A* expression by two *z*-scores (0.44log_10_ copies/ml lower VL) is comparable in magnitude to the effect of the presence of HLA-B*57 (0.41 log_10_ copies/ml lower VL in the same data set).

Taken together, these data support a model of increased *HLA-A* expression having a deleterious effect on HIV control through enhanced HLA-E expression that results in increased NKG2A-mediated NK (and/or T cell) inhibition, and impaired elimination of HIV-infected target cells.

HIV is capable of avoiding both T cell and NK cell recognition of infected host cells. HIV Nef-mediated reduction of HLA-A and -B (22) surface expression and Vpu-mediated reduction of HLA-C (23) likely serve to reduce antigen presentation and T cell killing of infected targets. These viral mechanisms occur posttranslationally (22, 23) and should not affect the contribution of HLA class I signal peptides to enhancing HLA-E expression. This in turn may serve to allow continued evasion of NK cell responses through enhanced NKG2A inhibition among those individuals with *HLA* haplotypes that provide ample signal peptide to bind HLA-E. HIV encodes a peptide (AISPRTLNA, AA9) that may further exploit the inhibitory effects of HLA-E, but discrepancies regarding the effects of this peptide on HLA-E expression, NKG2A binding, and NK cell killing have been reported (24, 25). NKG2A-expressing CD8 T cells are involved in antiviral responses (26), but the functional assays that we used are not appropriate for evaluating CD8^+^ T cell responses, and thus, we cannot rule out a role for CD8 T cells in the genetic data presented herein. Although NKG2C, an activating receptor that also binds HLA-E (27), may play some role in the pathway that we delineate, signaling through NKG2A dominates and overrides NKG2C signaling (28).

These data show that expression level variation participates in the complex patterns of *HLA* associations in HIV disease, a pattern recognized for class I in other species (29). Blockade of HLA-E:NKG2A–mediated inhibition in vivo is a therapeutic strategy being explored through clinical trials of an antibody against NKG2A (monalizumab) for treatment of rheumatoid arthritis (NCT02331875), cancer (NCT 02557516, NCT02643550, NCT02459301, NCT02671435), and stem-cell transplantation (NCT02921685), because a role for HLA-E–mediated immunosuppression is recognized in these disorders (30, 31). Our data suggest that antagonizing HLA-E/NKG2A interactions, perhaps in combination with other therapies, may provide benefit in HIV disease. This might be an attractive approach in HIV cure strategies. Genetic validation of NKG2A as a therapeutic target in additional diseases by testing for effects of *HLA-A* and *HLA-B* −*21* genotypes may rationalize the use of anti-NKG2A therapy in other disorders.

## Supplementary Material

Supp Figures 1-3

## Figures and Tables

**Fig. 1:**
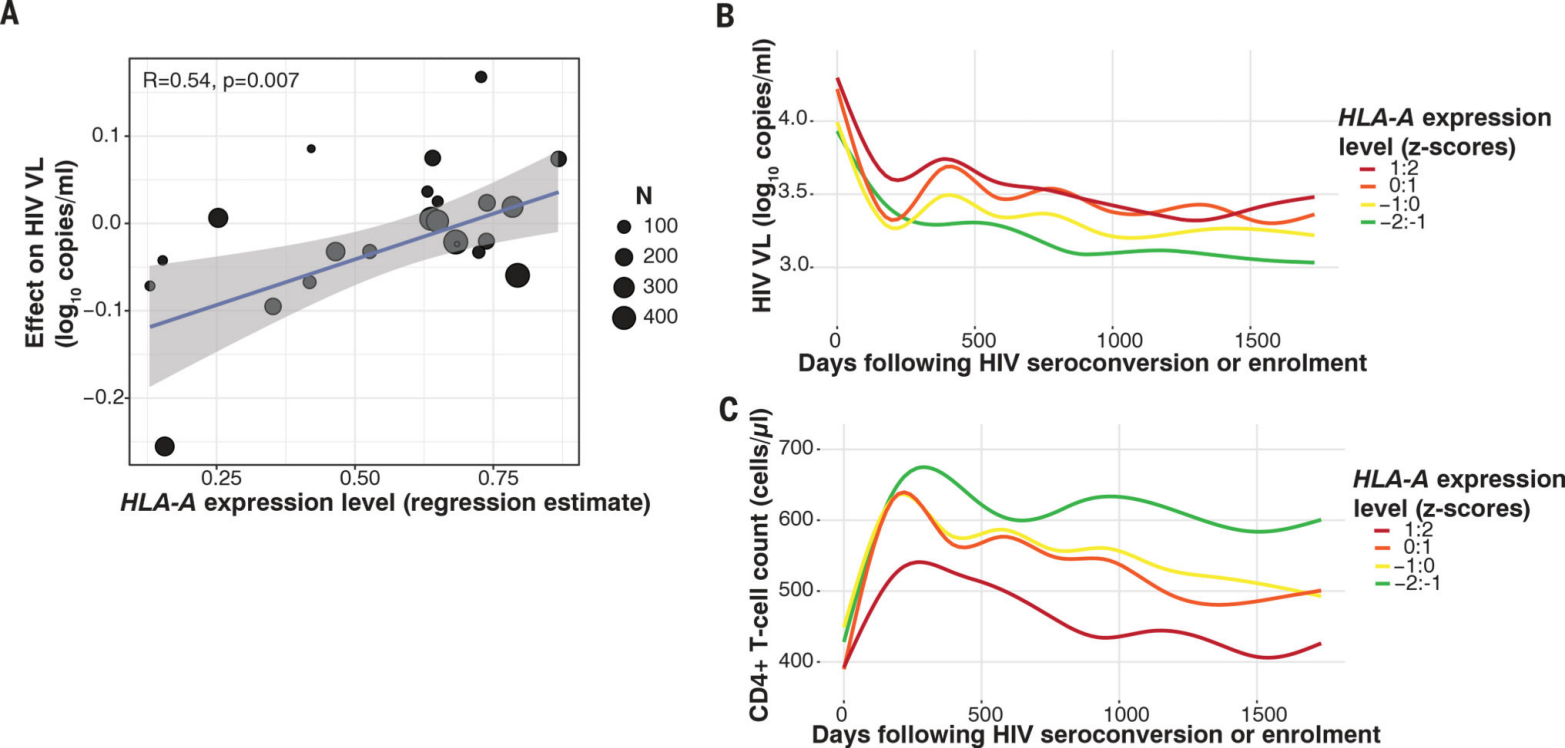
Elevated *HLA-A* expression levels are associated with increased HIV viremia and reduced CD4^+^ T cell counts (**A**) Data represent 2298 HIV-infected individuals from South Africa, Botswana, and Zambia, enrolled at 11 sites with cross-sectionally measured VLs. Each dot represents the average estimated expression level for a specific *HLA-A* allele by that allele’s reported effect on cross-sectional VL (11). A linear regression line is shown in blue with 95% confidence interval in gray. The size of each point is scaled by the number of contributing alleles; however, the correlation estimate is not weighted. (**B**) HIV viremia among 5818 HIV-infected adults and (**C**) CD4^+^ T cell counts among 2100 HIV-infected adults followed prospectively and grouped according to one-unit *z*-score change in *HLA-A* expression. VLs are plotted against time following seroconversion or date of enrollment (censored at ~5 years). In (B) and (C), lines are best fit (LOWESS lines) to unadjusted VL or CD4 counts.

**Fig. 2:**
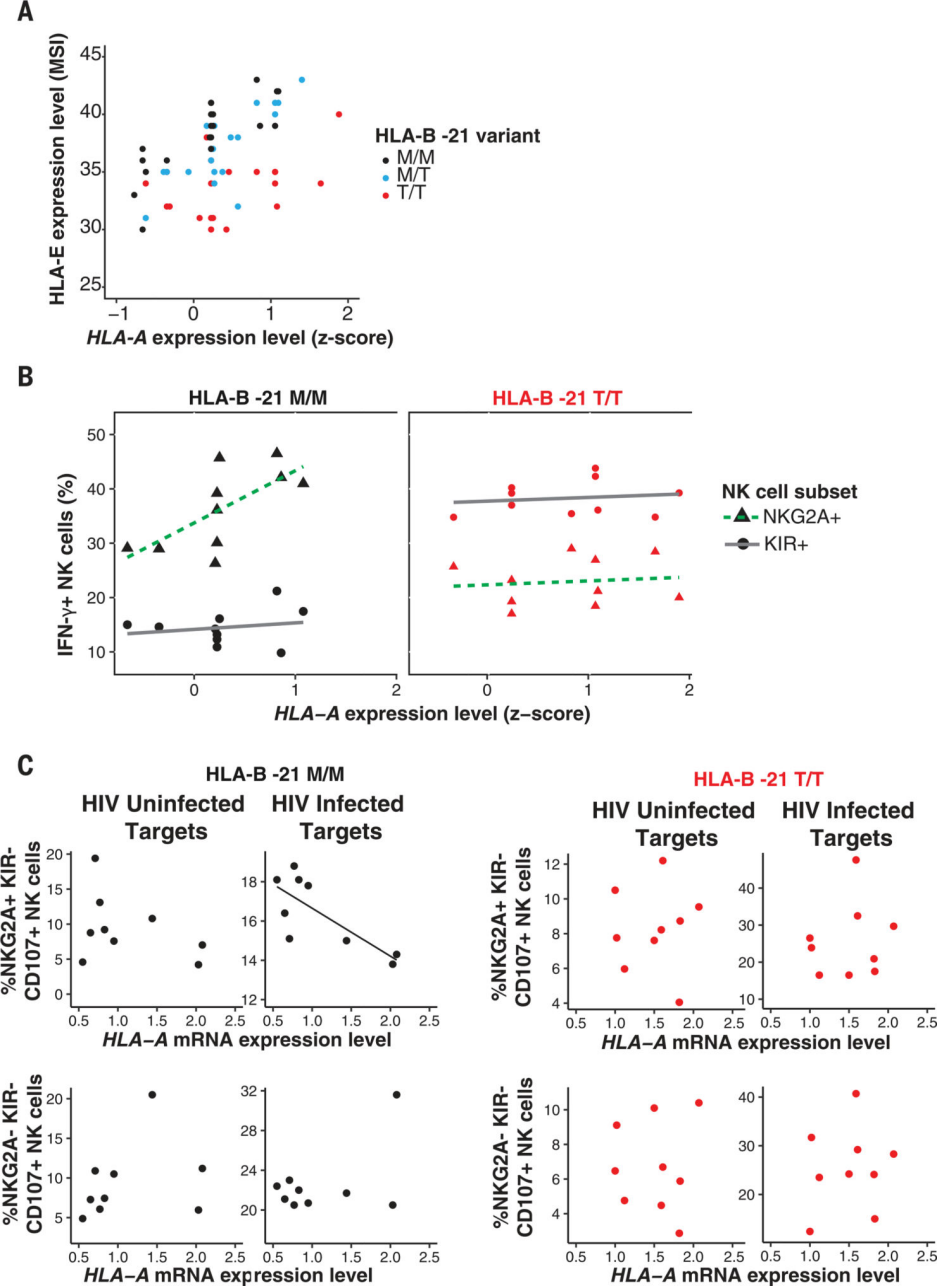
*HLA-A* expression and HLA-B −21M regulate HLA-E expression, resulting in biased licensing of NKG2A-expressing NK cells that are impaired in their killing of HIV-infected target cells (**A**) HLA-E expression according to *HLA-A* expression and HLA-B −21M in 58 HIV-uninfected donors. Each dot represents HLA-E expression levels (expressed as median signal intensity on a linear scale), as determined by CyTOF (15), and imputed *HLA-A* expression (*z*-score) (*R*_pearson_ = 0.43; 95% CI 0.20–0.62; *P* = 5 × 10^−4^). (**B**) NKG2A^+^ NK cell licensing varies by HLA-A expression and HLA-B −21M. Peripheral blood mononuclear cells (PBMCs) from 10 HLA-B −21M/M and 10 HLA-B −21T/T donors were coincubated with Raji cells pretreated with mouse antibody (2.5 µg/ml) against human CD20 for 6 hours to probe NK cell licensing and education. Each point represents the proportion of IFN-γ^+^ NK cells from each individual that are NKG2A^+^/KIR^−^ (triangles) or KIR^+^/NKG2A^−^ (circles) as a function of *HLA-A* expression. Dotted and solid lines show best fit lines for NKG2A^+^ and KIR^+^ subsets, respectively. The association between NK cell responsiveness and *HLA-A* expression for NKG2A^+^ NK cells in HLA-B −21M/M donors was *R*_pearson_ = 0.69 (95% CI 0.10–0.92), *P* = 0.03; all other correlations were not significant. (**C**) PBMCs from 9 HLA-B −21M/M and 9 HLA-B −21T/T donors were cocultured for 6 hours with autologous T cell blasts that were left uninfected or were infected with HIV [vesicular stomatitis virus G glycoprotein (VSV-G) pseudotyped NL4-3] and stained for CD107A, a marker of NK cell degranulation (see fig. S3 for gating strategy). *HLA-A* expression was formally measured in these T cell blasts by quantitative polymerase chain reaction and is expressed relative to β*2M* expression levels. Plots show individual proportions of NK cells expressing CD107a among NKG2A^+^KIR^−^ and NKG2A^−^KIR^−^ subsets. A best fit line is shown for significantly correlated observations. Red and black lines and dots denote TT and MM donors, respectively. The association between NKG2A^+^KIR^−^ NK cell response to HIV-infected target cells, and *HLA-A* expression in HLA-B −21M/M donors was *R*_pearson_ = –0.77 (95% CI –0.21 to –0.95), *P* = 0.02; all other correlations were not significant.

**Fig. 3:**
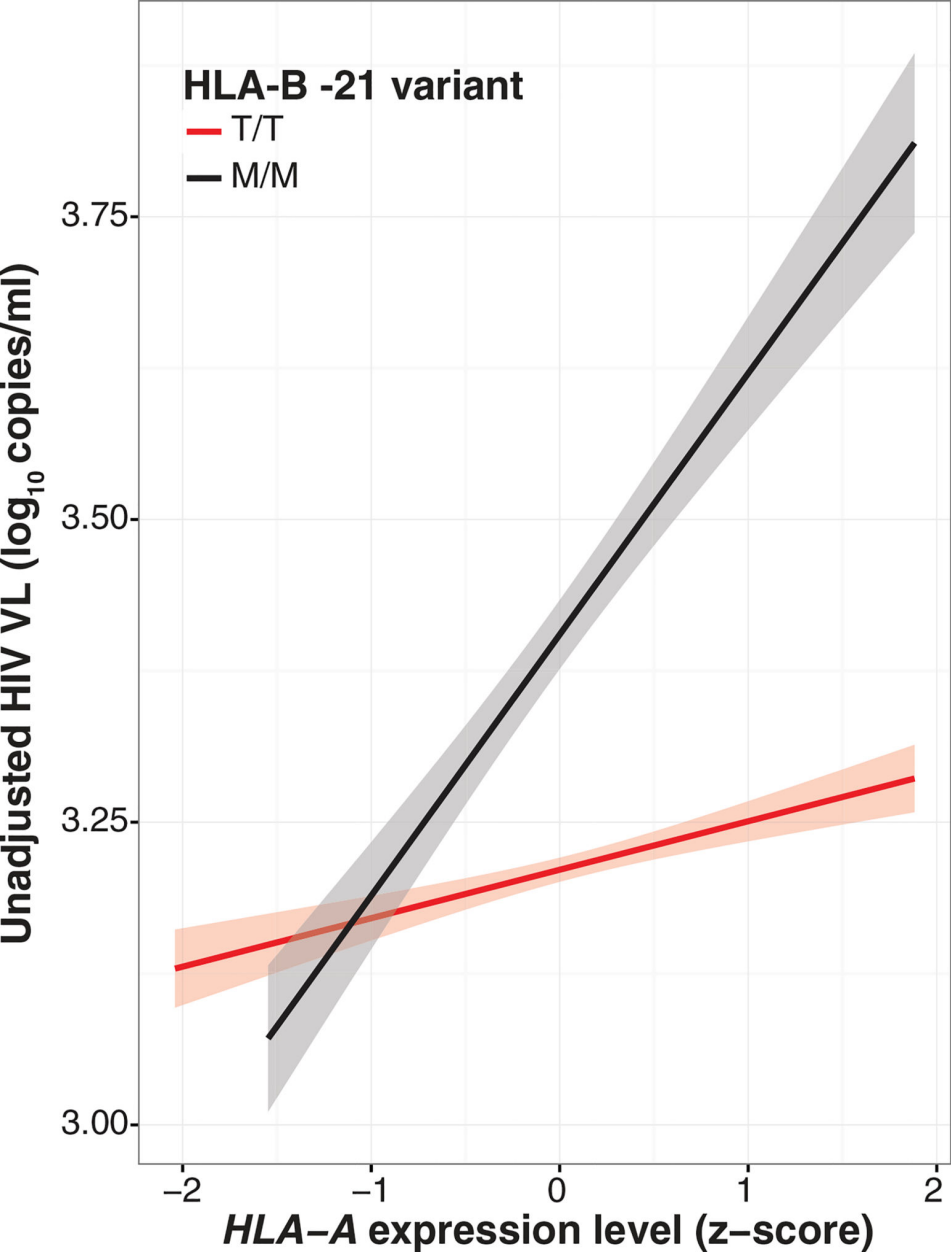
The effect of *HLA-A* expression on HIV VL is modified by *HLA-B* alleles encoding methionine at position −21 in the signal peptide The magnitude of effect (slope) of *HLA-A* expression on HIV viral load is stronger among individuals with *HLA-B* −21 MM (VL from 428 individuals, black line, VL_effect-MM_ = 0.22 log_10_ copies/ml, *P* = 1.5 × 10^−21^ adjusted for *HLA-A, -B*, and -*C*) compared with HLA-B TT (VL from 3071 individuals, red line, VL_effect-TT_ = 0.06 log_10_ copies/ml, *P* = 1.8 × 10^−9^ adjusted for *HLA-A, -B*, and -*C*). Interaction *P* = 5.3 × 10^−9^. Gray shading represents 95% CI of the linear estimate.

**Table 1:** *HLA-A* expression level is associated with impaired HIV control and is robust to multiple outcome definitions, and subset analyses across 9763 independent individuals of varying geographic and ethnic background Effect estimates denote the effect of one z-score (i.e. one standard deviation) increase in *HLA-A* expression on the outcome denoted.

Study	Outcome measure	Modeling approach	*n*	Effect estimate per *HLA-A z*-score increase	95% CI	*P*-value
**Cross-sectional discovery studies**						

**Pooled analysis of 2298 individuals from 11 African sites** (11). **Black individuals only.**	Viral load (log_10_ copies/ml)	Spearman correlation of VL effect and expression level for 23 *HLA-A* alleles	2298 volunteers	Spearman *R* = 0.54	NA‡	0.007

**Prospective validation studies with longitudinal follow-up**						

**Pooled analysis of 5818 individuals from six U.S. cohorts (ACTG, ALIVE, MACS, MHRP, Ragon, SCOPE) and one Swiss cohort (SWISS). Pooled data from 3442 white, 1497 black, 233 Hispanic, 60 Asian, 14 other, and 572 of mixed or other ancestry.**	Longitudinal viral load (VL)	Mixed effects-linear*				

	All individuals		62,843 VL in 5,818 volunteers	0.06 log_10_ copies/ml	0.05:0.08	4.4 × 10^−19^

	Known date of seroconversion		21,817 VL in 2,019 volunteers	0.06 log_10_ copies/ml	0.04:0.08	2.5 × 10^−9^

Mean viral load (mVL)	Mixed effects-linear*	5,818 mVL in 5,818 volunteers	0.14 log_10_ copies/x	0.10:0.18	9.3 × 10^−12^	

Controller/non-controller	Mixed effects-binomial*	2011 controller/2997 noncontroller	OR§ = 1.30	1.20:1.42	9.2 × 10^−11^	

CD4^+^ T cell count (cells/µl)	Mixed effects-linear*	56,415 CD4 counts in 2,100 volunteers	−37.8 cells/µl	−41.3:34.2	5.9 × 10^−94^	

**Prospective natural history validation studies**						

**Pooled analysis of 1159 individuals from five U.S. sites (ALIVE, MACS, MHCS, SFCCC and DCGCS). Pooled data from white, black, Hispanic or other ethnicities.**	Time to AIDS (CDC 1987)	Mixed effects-Cox*	1159 at-risk individuals, 433 events	HR‖ = 1.25	1.01:1.55	0.04

Time to CD4 <200 cells/µl	Mixed effects-Cox*	1159 at-risk individuals, 537 events	HR = 1.24	1.03:1.49	0.02	

**Reanalysis of broad HIV case-control genome-wide association study**						

**Pooled analysis of 3057 white, Hispanic, and black ethnicities.**	Controller/ non-controller	Logistic-regression†	737 controller/2300 noncontroller	OR = 1.29	1.14:1.45	2.7 × 10^−5^

* *HLA-A, -B*, and -*C* alleles, and timing of viral load measurements (for prospective studies) were taken into account by being coded as random effects.

† For GWAS analysis, population structure was adjusted for using the top five principal components.

‡ NA, not applicable.

§ OR, odds ratio.

‖ HR, hazard ratio.
