# Supplementary material for: Elevated HLA-A expression impairs HIV control through inhibition of NKG2A-expressing cells
Source: Science. Author manuscript; Available in PMC 2018 Jul 5. (PMC5933048; doi:10.1126/science.aam8825)
Supplement: Supp Figures 1-3 [file NIHMS949646-supplement-Supp_Figures_1-3.docx]

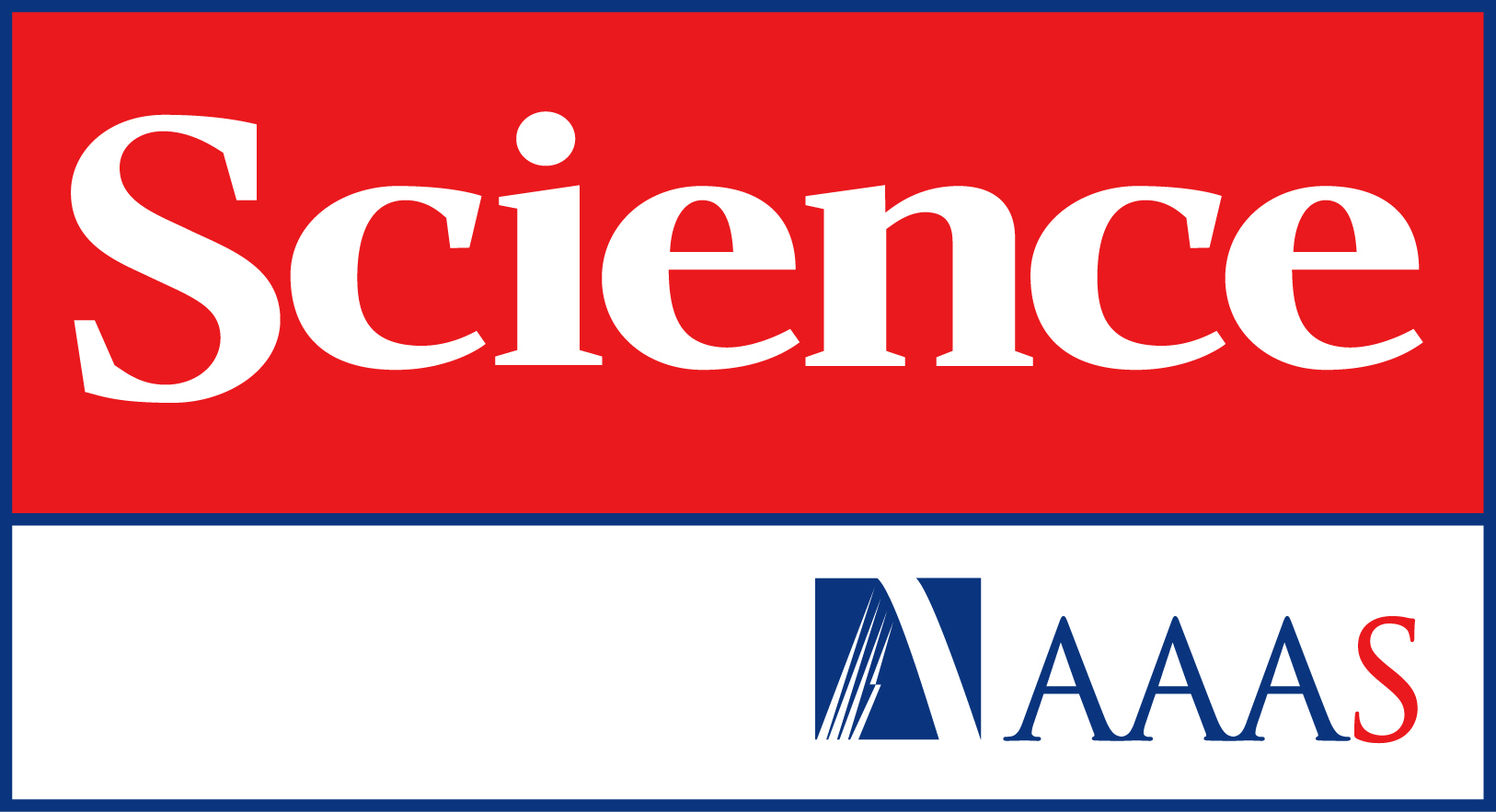


Supplementary Materials for

**Elevated *HLA-A* expression impairs HIV control through inhibition of Natural Killer cells**

Veron Ramsuran^1, 2, 3, 4, §^, Vivek Naranbhai^1, 2, 4, 5, §^, Amir Horowitz^6^, Ying Qi^1,2^, Maureen P. Martin^1^, Yuko Yuki^1^, Xiaojiang Gao^1^, Victoria Walker-Sperling^1^, Gregory Q. Del Prete^7^, Douglas K. Schneider^7^, Jeffrey D. Lifson^7^, Jacques Fellay^8^, Steven G. Deeks^9^, Jeffrey N. Martin^10^, James J. Goedert^11^, Steven M. Wolinsky^12^, Nelson L. Michael^13^, Gregory D. Kirk^14^, Susan Buchbinder^10,15^, David Haas^16^, Thumbi Ndung’u^2, 17^, Philip Goulder^17, 18^, Peter Parham^19^, Bruce D. Walker^2, 17, 20^, Jonathan M. Carlson^21^ & Mary Carrington^1,2 *^

Correspondence to: [carringm@mail.nih.gov](mailto:carringm@mail.nih.gov)

**This PDF file includes:**

Materials and Methods

SupplementaryText

Figs. S1 to S4

Table S1-S3

References S31-S48

Materials and Methods

Included cohorts

In total we included 9,763 adult HIV infected volunteers enrolled across 11 cross-sectional discovery, seven prospective and five natural history cohort studies as well as a pooled case-control genome-wide association study.

For discovery studies in non-Caucasian populations, we used published estimates of mean *HLA* allelic effects on viremia that were derived from 2,298 donors from 11 cohorts, described in detail by Carlson et al., 2016 (*11*). The 11 cohorts included individuals of Southern African descent from six locations; Durban - South Africa (*32*), Bloemfontein - South Africa (*33*), Kimberley - South Africa (*34*), Lusaka – Zambia (*35*), Gaborone – Botswana (*36*), and individuals of Southern African birth seeking care in the Thames Valley-United Kingdom (*34*). Estimates of effect on cross-sectionally measured VL were reported in the supplementary data (*11*).

For validation analyses of correlates of HIV viral control, we included 5,818 individuals enrolled in one of seven prospective studies and for whom we had ≥2 VL each: Adult Clinical Trials Group (ACTG, n=1,286) (*37*), AIDS Linked to the Intravenous experience (ALIVE, n=350) (*38*), Multicenter AIDS Cohort Study (MACS, n=1,667) (*39*), Military HIV Research Programme (MHRP, n=255) (*40*), The Ragon Institute of MGH, MIT and Harvard HIV Controller Study (n=1,714), Study of the Consequences of the Protease Inhibitor Era (SCOPE, n=404) (*41*), and the Swiss HIV Cohort Study (n=142). We considered pre-treatment VL measures only, and estimated the time post seroconversion (where known), or following the first date of enrolment. We defined HIV controllers as individuals with mVL<2,000 copies/ml and HIV non-controllers as individuals with HIV mVL>10,000 copies/ml and excluded (n=300) individuals with fewer than two viral load measures.

For survival analyses we included 1,159 donors from one of five studies: AIDS Linked to the Intravenous experience (ALIVE) (*38*), Multicenter AIDS Cohort Study (MACS) (*39*), Multicenter Hemophilia Cohort Study (MHCS) (*42*), San Francisco City Clinic Cohort (SFCCC) (*43*) or the DC Gay Cohort Study (DCGCS) (*44*) with prospective follow-up and known dates of seroconversion. Of the 1,159 donors, 705 (60.8%) were included in both longitudinal VL and survival analyses.

We note that several of the cohorts (ACTG, ALIVE, DCGS, SFCC, MACS and MHCS), were included in GWAS studies of HIV VL (*45*), but neither *HLA-A* expression nor the variant encoding HLA-B -21M/T could be identified because the former is not tagged by a single-nucleotide polymorphism, and the latter was not included on the chip. We therefore *HLA*-typed and inferred expression level for 3,057 participants of the International HIV Controllers GWAS study (*46*) who we also *HLA* typed. 1864 (61.0%) donors who were enrolled in the GWAS studies were also included in the longitudinal VL analyses above.

*HLA* typing

Individuals were genotyped at *HLA-A/B/C* by a PCR-SSOP (sequence-specific oligonucleotide probing) typing protocol or by PCR-SBT (sequence based typing) as recommended by the 13th International Histocompatibility Workshop(*47*) (<http://www.ihwg.org/tmanual/TMcontents.html>). Typing at four-digit resolution was achieved for all *HLA* class I alleles with the exceptions of *A*74:01/2, B*81, Cw*17* and *Cw*18*, that were determined to two-digit resolution only.

*HLA-E* genotyping was performed (n=3,831) by sequencing the region surrounding rs1264457A/G, which differentiates *HLA-E*01:01* (Arginine at position 107 of the mature protein) from *HLA-E*01:03* (Glycine at position 107 of the mature protein). PCR amplification and Sanger sequencing was performed using the primers reported by Paquay et al. (*48*) PCR primers used: 5’UTR (F): cagcgtcgccacgactcccgac; intron 5 (R): ggctcgtgtgtgtggatgg.

Sequencing primer (intron 2): agattcaccccaaggctg.

Measuring *HLA-A* mRNA expression levels

*HLA-A* mRNA expression levels were measured within two cohorts, FRESH (HIV negative, n=243) and SK (HIV infected – treatment naïve, n=162), using a quantitative PCR (qPCR) assay previously described (*7, 49*). Briefly, RNA and DNA was extracted from peripheral blood mononuclear cells (PBMCs) using the AllPrep DNA/RNA Mini Kit (Qiagen), RNA was treated with RNAse free DNAse to remove genomic DNA, and quantitated using HT RNA Lab Chip (Caliper, Life Sciences). A total of 1µg of RNA was used in a reverse transcription assay converting RNA to cDNA using the high capacity RNA to cDNA kit (Applied Bioscience). *HLA-A* and the house keeping gene *beta-2 microglobulin* (*β2M*) were amplified with Power SYBR green PCR master mix (Applied Biosystems) on the ABI7900HT machine (Applied Bioscience) using previously described PCR conditions(*49*). The *HLA-A* expression levels were calculated using the 2^-∆∆Ct^ method after normalizing to *β2M*.

Estimation of *HLA-A* expression

For the discovery analysis, we inferred the *HLA-A* expression of each four-digit allele reported previously (*11*), using regression estimates from measured expression in 244 black African individuals alone.

For all subsequent analyses (validation) that include individuals of varying ethnicity, we estimated *HLA-A* expression from *HLA-A* typing results using linear regression estimates from a prediction model. The model was built using mRNA measures of *HLA-A* from 436 HIV-uninfected Caucasian and African individuals combined, as we found no evidence for race-specific allele expression levels overall (Supplementary Table 1). We used two-digit resolution consistent with our previous report (*7*). The correlation between estimated and measured *HLA-A* expression was 0.79 (95% CI 0.74-0.82) where we trained the prediction model on a random sample of ~50% of individuals and tested prediction accuracy among the remainder in a 10,000-fold cross-validation.

We inferred HLA-B -21 amino acid variants and HLA-B serogroup using the aligned protein sequences available from the IMGT (*50*).

We used regression estimates from a previously reported study of HLA-C expression to estimate HLA-C expression (*1*) in order to verify the independence of the HLA-A and HLA-C effects.

HLA-E measurement and antibody-dependent cell-mediated cytotoxicity assays of NK cell responsiveness

HLA-E cell-surface expression was determined on bulk PBMC from each of 58 healthy adults with clone 3D12 (BioLegend) conjugated to 165Ho using a Maxpar labeling kit (Fluidigm, Inc). Clone 3D12 was one of a panel of 39 antibodies, as well as cisplatin (used as a cell viability reagent), as previously described (*15*).

All individuals had negative serological responses to cytomegalovirus using a clinically approved assay.

ADCC assays measuring activation of NK cells were performed by co-culturing PBMC from healthy donors with Raji cells for 6 hours. The Raji cells were pre-treated with mouse anti-human CD20 antibody [2.5ug/ml], which facilitates activation of NK cells independently of KIR or NKG2A, but rather through the Fc receptor. The antibodies targeted CD56-174Yb (clone HCD-56), NKG2A-171Yb (clone Z199), KIR2DL1-166Er (clone 143211), KIR2DL2/L3-149Sm (clone GL183), KIR3DL1-163Dy (clone DX9) and KIR3DL2-172Yb (clone DX31). NK cells were identified with a serial gating strategy as described(*15*). NKG2A+ NK cells were defined as NKG2A+KIR- and KIR+ NK cells were defined as KIR+NKG2A- NK cells.

The data were acquired with a CyTOF 2 instrument (Fluidigm Inc.) and analyzed using FlowJo software v9.4.8 (Tree Star Inc.) and Cytobank (Cytobank Inc.).

In vitro assessment of NK cell and CD8+ T-cell responses towards autologous HIV-infected T-cell blasts.

HIV-exposed uninfected donors from the FRESH (n=18) cohort were selected based on HLA-B -21 genotypes; 9 *HLA-B -21MM* and 9 *HLA-B -21TT*. Two vials of PBMCs were obtained from each donor. One vial per donor was thawed and stimulated with 30 U/ml of IL-2 and activated with 10ug/ml anti-CD3/CD28 Dynabeads (ThermoFisher). These cells were cultured overnight and divided into two subsets the next day. One subset was infected with NL4-3 (VSV-g) virus by spinoculation at 1,200xg for 1.5 hours at 37 degrees C, while the other subset was not infected. Both subsets were cultured in a CO_2_ incubator for a further 48 hours. HIV infection was measured by intracellular staining of HIV-1 p24 using anti-p24 PE (BD Biosciences) and was on average 46 to 78 %. The second vial of PBMC for each donor was thawed and stimulated with 200U/ml IL-2 and cultured overnight. Effectors (second vial of PBMC) and targets (uninfected and infected cultures) were cocultured at an effector to target ratio of 10:1. GolgiPlug (containing brefeldin A) and GolgiStop (containing monensin) (BD BioSciences) were added to the cultures at 1 μl/10^6^ cells and 0.75 μl/10^6^ cells respectively. Cultures were incubated for 6 hours before washing and staining with surface markers in a volume of 50 μl. Cells were stained with monoclonal antibodies directed against CD3 PacBlue (UCHT1), CD4 BV786 (SK3), CD8 APC-H7 (SK1; BD Biosciences), CD56 BV711 (HCD56), CD107a BV650 (H4A3; BioLegend), KIR2D PE (NKVFS1), NKG2C FITC (REA205; Miltenyi) and NKG2A APC (Z199; Beckman Coulter). The gating strategy for NK cells shown in Figure 2 is shown in Supplementary Fig. 3. CD107a+ cells were enumerated amongst cells gates as CD3-, CD56+ KIR- according to presence or absence of NKG2A. Data was acquired on a BD LSRFortessa™ and data was analyzed using FlowJo software v9.5.8 (Tree Star Inc.).

Regression modelling

Regression analyses were performed in R (v 3.3.1) using the following packages: lme4, ggplot2 and BioStrings.

Analyses of VL and CD4+ T-cell count were performed using the lmer function. We allowed for random effects due to each *HLA-A*, *-B* and *-C* allele, the time post enrolment and a correction for diploid *HLA* allele coding. We tested several alternative outcomes including the log_10_ transformed HIV VL at each timepoint, a geometric mean HIV VL (mVL) of all timepoints and the CD4+ T-cell count at each timepoint. For dichotomous outcomes (HIV controller vs. non-controller) we used a binomial null distribution. We calculated likelihood ratio p-values using the ANOVA function in R, to compare nested models fit under a maximum-likelihood scenario. The p-values reported are not adjusted for multiple comparisons.

For re-analysis of GWAS studies (*46*) we used the reported elite controller case-control status, reported principal components and formal *HLA*-typing results to perform logistic regression correcting for the first five principal components.

For survival analyses we used the coxme function and estimated the time to AIDS (CDC 1987 definition) or CD4+ T-cell count<200 cells/μl.

Supplementary Text

Extended Acknowledgements

This work was supported by federal funds from the National Cancer Institute, National Institutes of Health (NIH), [grant numbers HHSN261200800001E, U01AI068636, N02-CP-55504, R01-DA04334 and R01-DA12568, R37-AI067073], and the Collaboration for AIDS Vaccine Discovery of the Bill and Melinda Gates Foundation. Support was also provided by NIH funded Centers for AIDS Research (P30 AI060354, Harvard University Center for AIDS Research), which are supported by the following NIH co-funding and participating Institutes and Centers: NIAID, NCI, NICHD, NHLBI, NIDA, NIMH, NIDCR, NIA, FIC, OAR and South African Medical Research Council Flagship Award (grant number MRC-RFA- UFSP-01- 2013/UKZN-HIVEPI). Further support was provided by the Sub-Saharan African Network for TB/HIV Research Excellence (SANTHE) – a DELTAS Africa Initiative programme (grant number DEL-15-006). The content of this publication does not necessarily reflect the views or policies of the Department of Health and Human Services and Department of the Army, nor does mention of trade names, commercial products, or organizations imply endorsement by the US Government. This Research was supported in part by the Intramural Research Program of the NIH, Frederick National Lab, Center for Cancer Research.

Data in this manuscript were collected by the Adult Clinical Trials Group (ACTG) supported by an ACTG Network Leadership Grant Award. We gratefully acknowledge the SDMC, participating CRSs, and Specialty Laboratories of the ACTG. The content is solely the responsibility of the authors and does not necessarily represent the official views of the National Institute of Allergy and Infectious Diseases or the National Institutes of Health.

Data in this manuscript were collected by the Multicenter AIDS Cohort Study (MACS) and/or the Women's Interagency HIV Study (WIHS). The contents of this publication are solely the responsibility of the authors and do not represent the official views of the National Institutes of Health (NIH). MACS (Principal Investigators): Johns Hopkins University Bloomberg School of Public Health (Joseph Margolick), U01-AI35042; Northwestern University (Steven Wolinsky), U01-AI35039; University of California, Los Angeles (Roger Detels), U01-AI35040; University of Pittsburgh (Charles Rinaldo), U01-AI35041; the Center for Analysis and Management of MACS, Johns Hopkins University Bloomberg School of Public Health (Lisa Jacobson), UM1-AI35043. The MACS is funded primarily by the National Institute of Allergy and Infectious Diseases (NIAID), with additional co-funding from the National Cancer Institute (NCI). Targeted supplemental funding for specific projects was also provided by the National Heart, Lung, and Blood Institute (NHLBI), and the National Institute on Deafness and Communication Disorders (NIDCD). MACS data collection is also supported by UL1-TR000424 (JHU CTSA). Website located at [www.statepi.jhsph.edu/macs/macs.html](http://www.statepi.jhsph.edu/macs/macs.html).

The Swiss HIV Cohort Study is supported by the Swiss National Science Foundation (grant #148522). The data are gathered by the Five Swiss University Hospitals, two Cantonal Hospitals, 15 affiliated hospitals and 36 private physicians (listed in http://www.shcs.ch/180-health-care-providers). Members of the Swiss HIV Cohort Study: Aubert V, Battegay M, Bernasconi E, Böni J, Braun DL, Bucher HC, Calmy A, Cavassini M, Ciuffi A, Dollenmaier G, Egger M, Elzi L, Fehr J, Fellay J, Furrer H (Chairman of the Clinical and Laboratory Committee), Fux CA, Günthard HF (President of the SHCS), Haerry D (deputy of "Positive Council"), Hasse B, Hirsch HH, Hoffmann M, Hösli I, Kahlert C, Kaiser L, Keiser O, Klimkait T, Kouyos RD, Kovari H, Ledergerber B, Martinetti G, Martinez de Tejada B, Marzolini C, Metzner KJ, Müller N, Nicca D, Pantaleo G, Paioni P, Rauch A (Chairman of the Scientific Board), Rudin C (Chairman of the Mother & Child Substudy), Scherrer AU (Head of Data Centre), Schmid P, Speck R, Stöckle M, Tarr P, Trkola A, Vernazza P, Wandeler G, Weber R, Yerly S.

The authors declare no competing financial interests.

**Supplementary Fig. 1.** *HLA-A* expression varies across allotypes across a gradient that is no different among 243 HIV uninfected and 162 HIV infected donors, of Zulu ethnicity. *HLA-A* expression was measured by quantitative PCR and expressed relative to *β2M* mRNA using the 2−ΔΔCt method. The relative expression level is plotted for twice for each donor, once for each *HLA-A* allotype. Formal linear regression modelling did not identify any significant difference in allele-specific expression levels attributable to HIV infection, nor was HIV infection a significant interaction term with any *HLA-A* allotype. The mean expression level for each allotype is denoted by the black line. Allotypes with fewer than five individuals contributing were excluded.

**Supplementary Fig. 2.** *HLA-B* alleles with methionine at position -21 in the signal peptide (-21M) are almost exclusively of the Bw6 serogroup and on haplotypes with *HLA-C* alleles of the C1 group. Consequently, individuals with HLA-B -21MM have greater NKG2A-mediated education and conversely, those with TT have more KIR-mediated education.

**
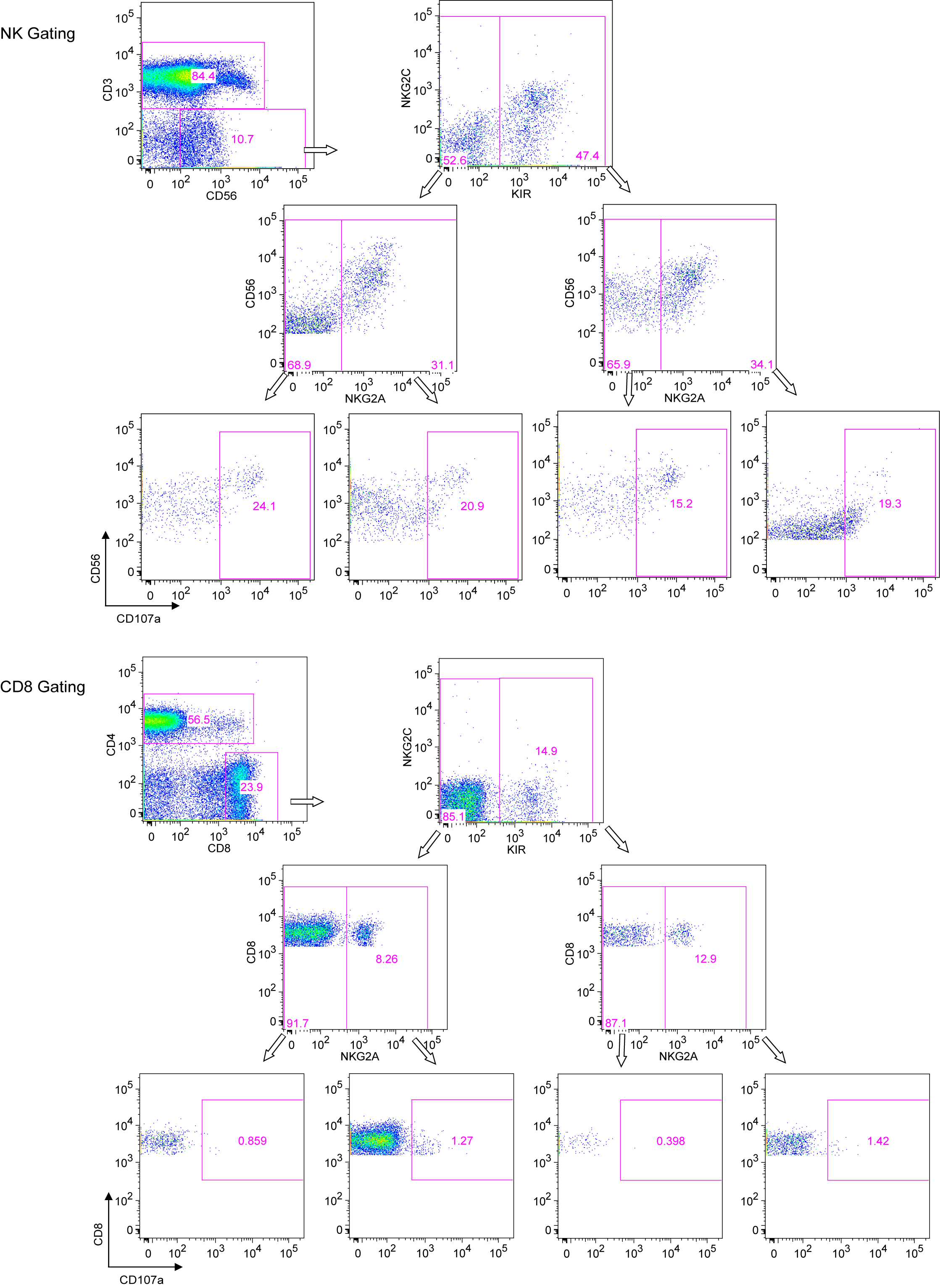
Supplementary Fig. 3. Gating strategy for NK cell coculture with autologous HIV infected cells at 10:1 effector:target ratio.**


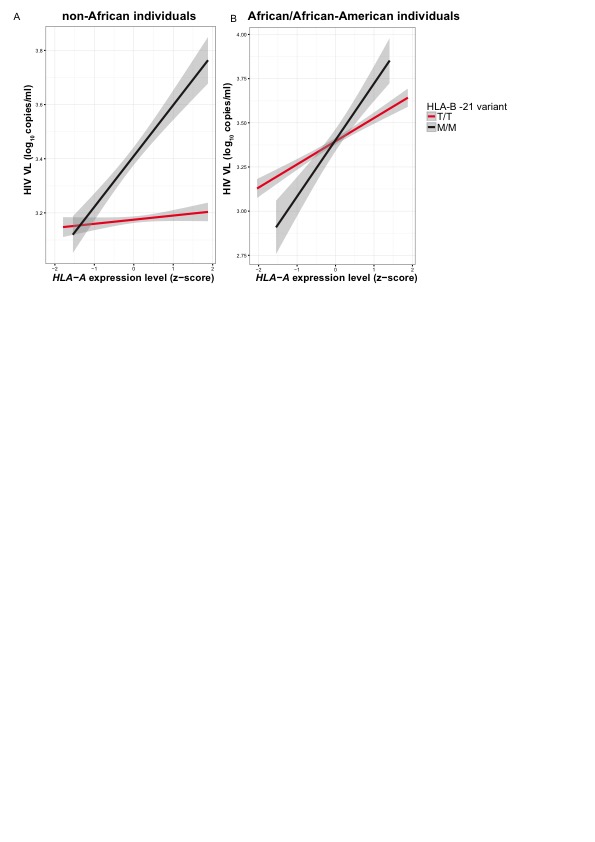


**Supplementary Fig. 4.** The effect of elevated *HLA-A* expression on HIV viremia is of larger magnitude in individuals with HLA-B -21MM than HLA-B -21TT, but the relative intercepts vary in Blacks and Whites, perhaps as a function of distinct *HLA* haplotypes and haplotype frequencies as have been reported (*15*).

Table S1.

Supplementary Table 1. *HLA-A* expression model estimates (z-score).

We estimated the *HLA-A* expression level for each *HLA-A* lineage among 436 HIV uninfected Caucasian and African individuals using a linear regression model. A z-score represents how many standard deviations (sd) an expression value is from the mean, hence a z-score of -1 represents an expression value one sd below the mean. Allelic variation explained 69.5% of the variance in *HLA-A* expression overall, allowing *HLA-A* expression levels to be inferred for each donor from their *HLA-A* typing results.

| ***HLA-A* Lineage** | **Expression estimate (z-score)** |
| --- | --- |
| *A*01* | 0.11 |
| *A*02* | 0.11 |
| *A*03* | -0.77 |
| *A*11* | 0.14 |
| *A*20* | -0.37 |
| *A*23* | 0.34 |
| *A*24* | 0.94 |
| *A*25* | -0.04 |
| *A*26* | 0.15 |
| *A*29* | 0.46 |
| *A*30* | 0.26 |
| *A*31* | -0.46 |
| *A*32* | -0.73 |
| *A*33* | -0.77 |
| *A*34* | 0.37 |
| *A*36* | 0.21 |
| *A*43* | -0.17 |
| *A*66* | 0.25 |
| *A*68* | 0.70 |
| *A*69* | 0.53 |
| *A*74* | -1.02 |
| *A*80* | -0.39 |

**Table S2.**

Supplementary Table 2. HLA-E expression is independently associated with *HLA-A* expression and HLA-B -21 variation, but not *HLA-E* genotype.

We measured HLA-E expression by CyTOF applied to PBMC from 58 healthy donors. Linear regression modeling was performed to estimate univariate (unadjusted) effects and multivariate (effects adjusting for each of the other covariables).

|  | **Univariate analysis** | | | **Multivariate analysis** | | |  |
| --- | --- | --- | --- | --- | --- | --- | --- |
|  | **Estimate (MSI units)** | **SE** | **p-value** | **Estimate (MSI units)** | **SE** | **p-value** | |
| *HLA-A* expression (per 1 z-score increase) | 2.53 | 0.69 | 5.26x10^-4^ | 3.22 | 0.51 | 4.19x10^-8^ | |
| HLA-B -21 M (per each addition -21M) | 2.28 | 0.48 | 1.43x10^-5^ | 2.57 | 0.41 | 6.51x10^-8^ | |
| *HLA-E* genotype (per each *E*01:03* allele) | 1.79 | 0.62 | 5.42x10^-3^ | 0.54 | 0.47 | 0.256 | |

**Table S3.**

Supplementary Table 3 HLA-A and HLA-B -21M, but not *HLA-E* genotype, are associated with HIV viremia .

Multivariate regression results of *HLA-A* expression, HLA-B -21M and *HLA-E* genotype on HIV viral load in a linear mixed effects model adjusted for *HLA-A*, *-B* and *-C* alleles as well as time post enrolment. We included 44,118 VL measurements from 3,831 volunteers.

|  | **Effect (log^10^ copies/ml)** | **SE** | **p-value** |
| --- | --- | --- | --- |
| *HLA-A* expression (per 1 z-score increase) | 0.06 | 0.008 | 3.39x10^-11^ |
| HLA-B -21 M (per each additional -21M) | 0.05 | 0.011 | 1.88x10^-5^ |
| *HLA-E* genotype (per each additional *E*01:03* allele) | <0.01 | 0.006 | 0.06 |

**References**

1. R. Apps *et al.*, *Science* **340**, 87 (Apr 5, 2013).

2. E. C. Ibrahim *et al.*, *The American journal of pathology* **162**, 501 (Feb, 2003).

3. E. W. Petersdorf *et al.*, *Blood* **124**, 3996 (Dec 18, 2014).

4. E. W. Petersdorf *et al.*, *The New England journal of medicine* **373**, 599 (Aug 13, 2015).

5. P. Raj *et al.*, *eLife* **5**, (2016).

6. W. T. Wissemann *et al.*, *American journal of human genetics* **93**, 984 (Nov 7, 2013).

7. V. Ramsuran *et al.*, *Human molecular genetics* **24**, 4268 (Aug 1, 2015).

8. R. Apps *et al.*, *Journal of immunology* **194**, 3594 (Apr 15, 2015).

9. S. Kulkarni *et al.*, *Nature* **472**, 495 (Apr 28, 2011).

10. N. Vince *et al.*, *American journal of human genetics* **1**, 1353 (2016).

11. J. M. Carlson *et al.*, *Nat Med* **22**, 606 (Jun, 2016).

12. F. Pereyra *et al.*, *Science* **330**, 1551 (Dec 10, 2010).

13. V. M. Braud *et al.*, *Nature* **391**, 795 (Feb 19, 1998).

14. N. Lee *et al.*, *Proceedings of the National Academy of Sciences of the United States of America* **95**, 5199 (Apr 28, 1998).

15. A. Horowitz *et al.*, *Science Immunology* **1**, (2016).

16. N. Lee, D. R. Goodlett, A. Ishitani, H. Marquardt, D. E. Geraghty, *Journal of immunology* **160**, 4951 (May 15, 1998).

17. V. Ramsuran *et al.*, *Journal of immunology* **15**, 2320 (2017).

18. R. K. Strong *et al.*, *J Biol Chem* **278**, 5082 (Feb 14, 2003).

19. M. T. Orr, L. L. Lanier, *Cell* **142**, 847 (Sep 17, 2010).

20. A. M. Merino *et al.*, *Clin Exp Immunol* **174**, 414 (Dec, 2013).

21. P. Brodin, K. Karre, P. Hoglund, *Trends Immunol* **30**, 143 (Apr, 2009).

22. G. B. Cohen *et al.*, *Immunity* **10**, 661 (Jun, 1999).

23. R. Apps *et al.*, *Cell Host Microbe* **19**, 686 (May 11, 2016).

24. Z. B. Davis *et al.*, *PLoS Pathog* **12**, e1005421 (Feb, 2016).

25. J. Nattermann *et al.*, *Antivir Ther* **10**, 95 (2005).

26. J. M. Moser, J. Gibbs, P. E. Jensen, A. E. Lukacher, *Nat Immunol* **3**, 189 (Feb, 2002).

27. H. Wada, N. Matsumoto, K. Maenaka, K. Suzuki, K. Yamamoto, *Eur J Immunol* **34**, 81 (Jan, 2004).

28. V. Beziat *et al.*, *Blood* **117**, 4394 (Apr 21, 2011).

29. J. Kaufman, H. Volk, H. J. Wallny, *Immunol Rev* **143**, 63 (Feb, 1995).

30. L. Wieten, N. M. Mahaweni, C. E. Voorter, G. M. Bos, M. G. Tilanus, *Tissue Antigens* **84**, 523 (Dec, 2014).

31. R. T. Manguso *et al.*, *Nature* **547**, 413 (Jul 27, 2017).

**Supplementary references**

32. J. M. Carlson *et al.*, *Science* **345**, 1254031 (Jul 11, 2014).

33. K. H. Huang *et al.*, *Antivir Ther* **14**, 975 (2009).

34. P. C. Matthews *et al.*, *J Immunol* **186**, 5675 (May 15, 2011).

35. M. C. Kempf *et al.*, *J Acquir Immune Defic Syndr* **47**, 116 (Jan 1, 2008).

36. R. L. Shapiro *et al.*, *N Engl J Med* **362**, 2282 (Jun 17, 2010).

37. D. W. Haas *et al.*, *HIV Clin Trials* **4**, 287 (Sep-Oct, 2003).

38. D. Vlahov *et al.*, *JAMA* **279**, 35 (Jan 7, 1998).

39. J. Phair *et al.*, *J Acquir Immune Defic Syndr* **5**, 490 (1992).

40. K. Pelak *et al.*, *J Infect Dis* **201**, 1141 (Apr 15, 2010).

41. B. Emu *et al.*, *J Virol* **79**, 14169 (Nov, 2005).

42. J. J. Goedert *et al.*, *N Engl J Med* **321**, 1141 (Oct 26, 1989).

43. S. P. Buchbinder, M. H. Katz, N. A. Hessol, P. M. O'Malley, S. D. Holmberg, *AIDS* **8**, 1123 (Aug, 1994).

44. J. J. Goedert *et al.*, *Am J Epidemiol* **121**, 629 (May, 1985).

45. P. J. McLaren *et al.*, *Proceedings of the National Academy of Sciences of the United States of America* **112**, 14658 (Nov 24, 2015).

46. H. I. V. C. S. International *et al.*, *Science* **330**, 1551 (Dec 10, 2010).

47. M. P. Martin *et al.*, *Nat Genet* **39**, 733 (Jun, 2007).

48. M. M. Paquay, J. Schellekens, M. G. Tilanus, *Tissue antigens* **74**, 514 (Dec, 2009).

49. S. Kulkarni *et al.*, *Proceedings of the National Academy of Sciences of the United States of America* **110**, 20705 (Dec 17, 2013).

50. J. Robinson *et al.*, *Nucleic Acids Res* **43**, D423 (Jan, 2015).
